# The flavonoid galangin is an inhibitor of CYP1A1 activity and an agonist/antagonist of the aryl hydrocarbon receptor

**DOI:** 10.1038/sj.bjc.6690216

**Published:** 1999-03

**Authors:** H P Ciolino, G C Yeh

**Affiliations:** Cellular Defense and Carcinogenesis Section, Basic Research Laboratory, Division of Basic Sciences, National Cancer Institute–Frederick Cancer Research and Development Center, Frederick, MD, 21702-1201, USA

**Keywords:** galangin, flavonoid, DMBA, CYP1A1, EROD

## Abstract

The effect of the dietary flavonoid galangin on the metabolism of 7,12-dimethylbenz[*a*]anthracene (DMBA), the activity of cytochrome P _450_ 1A1 (CYP1A1), and the expression of *CYP1A1* in MCF-7 human breast carcinoma cells was investigated. Galangin inhibited the catabolic breakdown of DMBA, as measured by thin-layer chromatography, in a dose-dependent manner. Galangin also inhibited the formation of DMBA-DNA adducts, and prevented DMBA-induced inhibition of cell growth. Galangin caused a potent, dose-dependent inhibition of CYP1A1 activity, as measured by ethoxyresorufin-*O*-deethylase activity, in intact cells and in microsomes isolated from DMBA-treated cells. Analysis of the inhibition kinetics by double-reciprocal plot demonstrated that galangin inhibited CYP1A1 activity in a non-competitive manner. Galangin caused an increase in the level of CYP1A1 mRNA, indicating that it may be an agonist of the aryl hydrocarbon receptor, but it inhibited the induction of CYP1A1 mRNA by DMBA or by 2,3,5,7-tetrachlorodibenzo-*p*-dioxin (TCDD). Galangin also inhibited the DMBA- or TCDD-induced transcription of a reporter vector containing the *CYP1A1* promoter. Thus, galangin is a potent inhibitor of DMBA metabolism and an agonist/antagonist of the AhR, and may prove to be an effective chemopreventive agent. © 1999 Cancer Research Campaign


*British Journal of Cancer* (1999) **79**(9/10), 1340–1346
© 1999 Cancer Research Campaign
Article no. bjoc.1998.0216

### The flavonoid galangin is an inhibitor of CYP1A1 activity and an agonist/antagonist of the aryl hydrocarbon receptor

**HP Ciolino and GC Yeh**

Cellular Defense and Carcinogenesis Section, Basic Research Laboratory, Division of Basic Sciences, National Cancer Institute–Frederick Cancer Research and Development Center, NIH, Frederick, MD 21702-1201, USA

**Summary** The effect of the dietary flavonoid galangin on the metabolism of 7,12-dimethylbenz[*a*]anthracene (DMBA), the activity of cytochrome $P_{450}$ 1A1 (CYP1A1), and the expression of *CYP1A1* in MCF-7 human breast carcinoma cells was investigated. Galangin inhibited the catabolic breakdown of DMBA, as measured by thin-layer chromatography, in a dose-dependent manner. Galangin also inhibited the formation of DMBA-DNA adducts, and prevented DMBA-induced inhibition of cell growth. Galangin caused a potent, dose-dependent inhibition of CYP1A1 activity, as measured by ethoxyresorufin-*O*-deethylase activity, in intact cells and in microsomes isolated from DMBA-treated cells. Analysis of the inhibition kinetics by double-reciprocal plot demonstrated that galangin inhibited CYP1A1 activity in a non-competitive manner. Galangin caused an increase in the level of CYP1A1 mRNA, indicating that it may be an agonist of the aryl hydrocarbon receptor, but it inhibited the induction of CYP1A1 mRNA by DMBA or by 2,3,5,7-tetrachlorodibenzo-*p*-dioxin (TCDD). Galangin also inhibited the DMBA- or TCDD-induced transcription of a reporter vector containing the *CYP1A1* promoter. Thus, galangin is a potent inhibitor of DMBA metabolism and an agonist/antagonist of the AhR, and may prove to be an effective chemopreventive agent.

**Keywords:** galangin; flavonoid; DMBA; CYP1A1; EROD

Numerous epidemiological studies have demonstrated that diets rich in fruits and vegetables are protective against several forms of cancer (Steinmetz and Potter, 1996), and much attention has been focused on whether naturally occurring dietary components can modify the mutagenic and carcinogenic effects of environmental carcinogens. 7,12-dimethylbenz[*a*]anthracene (DMBA), a model compound which induces mammary tumorigenesis in rodents (Huggins et al, 1961), is a member of one such class of carcinogen, the aryl hydrocarbons. Aryl hydrocarbons bind and activate the aryl hydrocarbon receptor (AhR), which induces the transcription of a number of genes (Rowlands and Gustafsson, 1997), including the cytochrome $P_{450}$ 1A (CYP1A) enzyme family. CYP1As mediate the oxidative catabolism of aryl hydrocarbons, generating genotoxic metabolites which can bind specific residues of DNA, introducing mutations in key genes and resulting in cellular transformation (Dipple, 1995). Inhibition of the metabolic activation of carcinogens, either through inhibition of the AhR-mediated signal transduction pathway or direct enzyme inhibition, is believed to be an important mechanism in chemoprevention.

Flavonoids are among the most abundant of phytochemicals and much attention has been focused recently on the effect of flavonoids on chemically induced in vivo models of carcinogenesis. Flavonoids consist of a diverse group of polyphenolic derivatives of benzo-γ-pyrone that are ubiquitous in foods of plant origin such as vegetables, fruit, tea and wine (Formica and Regelson, 1995). Dietary intake of flavonoids has been estimated at 1 g per day (Kuhnau, 1976), but recent studies have indicated that consumption varies widely (Hollman and Katan, 1997; Hollman et al, 1997). In vivo studies have shown that some flavonoids are effective in preventing chemically induced cancer in rodents, including DMBA-induced mammary cancer (Verma et al, 1988; Lamartiniere et al, 1995). Flavonoids exert a multiplicity of biochemical actions that are believed to be an important part of the chemopreventive effect of plant-based diets. Various flavonoids are potent antioxidants (Kono et al, 1997; Noda et al, 1997), inhibit lipoxygenase and cyclooxygenase (Mirzoeva and Calder, 1996), affect the activity of several hepatic enzymes involved in activation and detoxification of carcinogens (Canivenc-Lavier et al, 1996), inhibit cellular proliferation (Tsyrlov et al, 1994; Siess et al, 1995; Moon et al, 1998), and induce apoptosis of tumour cells (Csokay et al, 1997). Galangin, a member of the flavonol class of flavonoids, is present in high concentrations in *Alpina officinarum* (common name: China or India root), which has been used as a spice and as a herbal medicine for a variety of ailments in Asia for centuries. Galangin has been shown to inhibit the proliferation of breast tumour cells (So et al, 1996, 1997), and to inhibit the cytochrome $P_{450}$-dependent hydroxylation of the aryl hydrocarbon benzo[*a*]pyrene in human liver microsomes (Buening et al, 1981).

For this report we have examined the effects of galangin on the carcinogen activation pathway mediated by the AhR. We have used the MCF-7 human breast carcinoma cell line because it is derived from the target tissue of DMBA, the mammary epithelium, and because AhR function and carcinogen activation has been well characterized in these cells (Christou et al, 1994; Moore et al, 1994; Dohr et al, 1995; Wang et al, 1995). Furthermore, a recent study has demonstrated that MCF-7 cells are similar to normal human mammary epithelial cells with regard to AhR

*Received 26 May 1998*
*Revised 16 September 1998*
*Accepted 22 September 1998*

*Correspondence to:* HP Ciolino, BRL, NCI-FCRDC, Building 560/Rm 12-05, PO Box B, Frederick, MD 21702-1201, USA

expression and CYP1A1 activity (Larson et al, 1998). We demonstrate that galangin inhibits the activation of DMBA by inhibiting AhR function and CYP1A1 enzyme activity, and thus may be a promising candidate for in vivo chemoprevention study.

### MATERIALS AND METHODS

### Materials

Except as noted, all chemicals were from Sigma (St Louis, MO, USA). All culture vessels were from CoStar (Cambridge, MA, USA). Galangin (Aldrich, Milwaukee, WI, USA) was dissolved in dimethylsulphoxide (DMSO) and stored at – 20°C.

### Cell culture

MCF-7 cells (American Type Culture Collection, Rockville, MD, USA) were grown in RPMI-1640 supplemented with 2 mM glutamine and 10% fetal bovine serum (all from BioFluids, Rockville, MD, USA). Cells were sub-cultured weekly using 0.25% trypsin/0.05% EDTA (BioFluids). All experiments were carried out at 37°C and 5% carbon dioxide on confluent cells in growth medium, except where indicated.

### Measurement of DMBA metabolism

All steps were carried out in the dark or under yellow light. MCF-7 cells in 175 $cm^2$ flasks were incubated in 12 ml of growth medium containing 0.1 μg $ml^{-1}$ [$^3$H]DMBA (Amersham, Arlington Heights, IL, USA) in the presence of DMSO (control), 1 μM, or 10 μM galangin. After 24 h, 1 ml of the medium was removed and extracted with 1 ml ethyl acetate. [$^3$H]DMBA was separated from metabolites by thin-layer chromatography. A total of 5 μl of the organic phase was applied to a 20 × 20 $cm^2$ silica thin-layer chromatography sheet with fluorescent indicator (Kodak, Rochester, NY, USA). Chromatography was performed in n-hexane for 80 min. The sheet was dried and 12 1.7-cm strips were cut, placed in 20 ml BSC-NA organic scintillation fluid (Amersham) and counted. The parent compound migrated to the seventh and eighth strips while the metabolites migrated to the first two strips (counted from the point of application). Spontaneous decomposition of [$^3$H]DMBA was determined by incubating flasks without cells under the same conditions. The amount of metabolites formed in blank flasks (20 ± 0.58%; $n$ = 3) was subtracted from the other incubations.

### Measurement of DMBA-DNA adduct formation

Confluent cultures of MCF-7 cells in 75 $cm^2$ flasks were exposed to 0.1 μg $ml^{-1}$ [$^3$H]DMBA in the presence of the indicated concentrations of galangin for 8 h. Cells were washed twice with cold phosphate-buffered saline (PBS), trypsinized and pelleted. Nuclei were isolated by incubating the cells for 10 min on ice in 10 mM Tris-HCl, pH 7.5, with 320 mM sucrose, 5.0 mM magnesium chloride and 1% Triton X-100. The nuclei were pelleted by centrifugation at 800 ***g*** for 10 min at 4°C, and this digestion was repeated once. Nuclei were then lysed with 1% sodium dodecyl sulphate (SDS) in 0.5 M Tris, 20 mM EDTA and 10 mM NaCl, pH 9.0, followed by treatment with 20 mg $ml^{-1}$ proteinase K for 6 h at 48°C. Protein and peptides were precipitated by the addition of NaCl to final concentration of 1 M and centrifuged at 500 ***g*** for 30 min at 4°C. Genomic DNA was then isolated from supernatant by repeated ethanol precipitation as described in Miller et al (1988). DNA isolated by this method exhibited a 260/280 ratio of > 1.9. The amount of DNA was measured by spectrophotometry and the adducts were quantified by liquid scintillation counting in Ecoscint A (National Diagnostics, Atlanta, GA, USA).

### Measurement of cell growth

MCF-7 cells were plated out in 24-well plates at 25 000 cell per well and allowed to attach for 24 h. DMBA was added at the indicated concentrations with either DMSO (control) or 5.0 μM galangin in fresh media. Total cell growth was assayed after 3 days using sulphorhodamine (Rubinstein et al, 1990).

### CYP1A1 activity in intact MCF-7 cells

MCF-7 cells in 24-well plates were treated with 1 ml growth medium containing 1 μM DMBA for 24 h in the presence of DMSO or the indicated concentrations of galangin. At the end of the incubation, the medium was removed and the wells were washed two times with fresh medium. Ethoxyresorufin-*O*-deethylase (EROD) activity, which is a specific assay of the bioactivation capacity of CYP1A1, was determined in intact cells as described in Kennedy and Jones (1994) using 5 μM ethoxyresorufin (ETRF) in growth medium as a substrate in the presence of 1.5 mM salicylamide to inhibit conjugating enzymes (Lubinski et al, 1994). The assay was carried out at 37°C. The fluorescence of resorufin generated by the conversion of ETRF by CYP1A1 was measured every 10 min for 60 min in a CytoFlor II multiwell fluorescence plate reader (PerSeptive Biosystems, Framingham, MA, USA), with an excitation of 530 nm and emission at 590 nm.

### Microsomal CYP1A1 activity

MCF-7 cells were treated with 1 μM DMBA for 24 h to induce CYP1A1 EROD activity. Microsomes were isolated as follows: the cells were washed once with PBS, trypsinized and pelleted by centrifugation at 800 ***g*** for 10 min at 4°C. The pellet was washed in PBS, repelleted, then resuspended in 0.25 M sucrose, 10 mM Tris-HCl, pH 7.5, with protease inhibitors (100 μg $ml^{-1}$ phenylmethylsulphonylfluoride, 300 μg $ml^{-1}$ EDTA, 0.5 μg $ml^{-1}$ leupeptin, 0.5 μg $ml^{-1}$ aprotinin and 0.7 μg $ml^{-1}$ Pepstatin A). The cells were sonicated for 30 s on ice using a Branson Sonifier at setting 2. The sonicate was centrifuged at 10 000 ***g*** for 10 min at 4°C and the supernatant was centrifuged at 500 000 ***g*** for 15 min at 4°C. The resulting microsomal pellet was resuspended in the above buffer and the protein assayed by the Bradford method (1976). Aliquots of microsomes were stored at – 80°C. CYP1A1 activity was determined by EROD assay in the following manner: for Figure 5A, 10 μg of microsomes were brought up to 100 μl with PBS, pH 7.2, 400 nM ETRF was added, along with DMSO or the indicated concentrations of galangin. The reaction was initiated by the addition of 250 μM NADPH. The reaction mixture was transferred to a 96-well plate and EROD activity was determined in a Cytoflor II Fluorescence Plate Reader as described above. For Figure 5B, 250 μM NADPH and 100–1600 nM ETRF were added to 3 ml of PBS, pH 7.2, 410 μl aliquots were removed to which DMSO or the indicated concentration of galangin were

© Cancer Research Campaign 1999

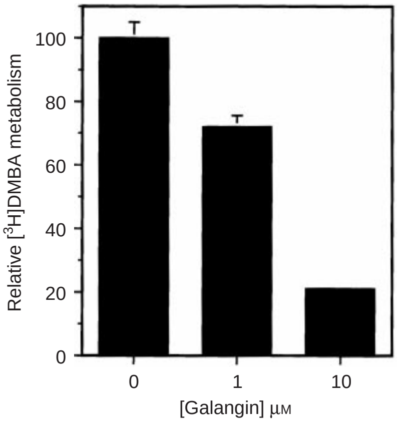

**Figure 1** Effect of galangin on the metabolic catabolism of [$^3$H]DMBA. MCF-7 cells were exposed to 0.1 μg ml$^{-1}$ [$^3$H]DMBA in the presence of DMSO (vehicle control), 1 μM, or 10 μM galangin for 24 h. [$^3$H]DMBA and metabolites were extracted and analysed by thin-layer chromatography. Each bar represents the mean of 3 determinations ± standard error (s.e.). DMBA metabolism in cultures treated with galangin was statistically different from controls ($P < 0.05$)

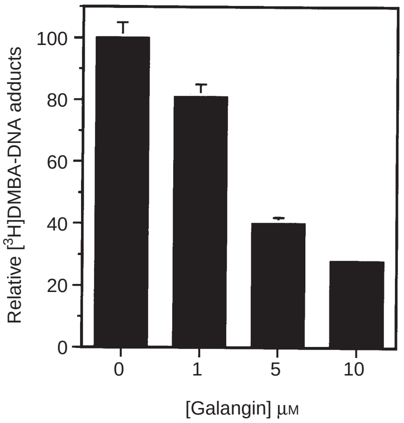

**Figure 2** Effect of galangin on [$^3$H]DMBA-DNA adduct formation. MCF-7 cells were incubated with 0.1 μg ml$^{-1}$ [$^3$H]DMBA in the presence of DMSO, or the indicated concentrations of galangin for 8 h. DNA was extracted and analysed for incorporation of [$^3$H]DMBA metabolites as described. Each bar represents the mean of 4 determinations ± s.e. Adduct formation in all galangin-treated samples was statistically different from controls ($P < 0.05$)

added. The reaction was initiated by the addition of 45 μg of microsomal protein (final volume was 450 μl) and gently vortexed. Four 100-μl aliquots (10 μg per assay) of each were removed, placed in a 96-well plate and assayed as above. A standard curve was constructed using resorufin.

### Reverse transcription polymerase chain reaction (RT-PCR)

Confluent MCF-7 cells were treated with a combination of galangin and 1 nM 2,3,7,8-tetrachlorodibenzo-*p*-dioxin (TCDD) or 250 nM DMBA for 6 h, or with galangin alone for 24 h. The cells were washed twice with PBS and total RNA was isolated using Trizol reagent (GibcoBRL, Gaithersburg, MD, USA). Semi-quantitative RT-PCR for CYP1A1 and glyceraldehyde-3-phosphate dehydrogenase (GPDH) was performed in the presence of 1.5 μCi [$^{32}$P]dATP (DuPont/NEN, Wilmington, DE, USA) using the primer sequences and conditions of Dohr et al (1995). cDNA was synthesized from 10 μg total RNA using a Stratagene RT-PCR kit (LaJolla, CA, USA) as instructed. The optimum cycle number that fell within the exponential range of response for both CYP1A1 (23 cycles) and GPDH (19 cycles) was used. Following PCR, 5 μl of high-density sample buffer was added to the samples and they were subjected to electrophoresis on a 10% Tris–borate–EDTA (TBE) gel in 1 × TBE running buffer (all components from Novex, San Diego, CA, USA). The gel was dried and the results were visualized and quantified on a BioRad GS-363 Molecular Imaging System (Hercules, CA, USA). Graphs of the resulting data were generated by normalizing CYP1A1 to GPDH.

### CAT–β-galactosidase assays

MCF-7 cells were plated at 60 000 cells per well in 24-well plates. After 24 h the cells were transiently transfected with 12.0 μg of a chloramphenicol acetyltransferase (CAT) reporter vector containing the full length rat *CYP1A1* promoter (pMC6.3K; Sogawa et al, 1986) using LipofectAmine (GibcoBRL) as directed. To control for transfection efficiency, the cells were co-transfected with 1.0 μg of pCMZ vector containing β-galactosidase (Clonetech Labs, Palo Alto, CA, USA). After a further 24 h, the cells were treated with 1 nM TCDD or 250 nM DMBA in the presence of DMSO (control) or galangin for 6 h. The amount of CAT transcription was determined using an ELISA assay (Boehringer Mannheim, Indianapolis, IN, USA) as directed. Activity of β-galactosidase was determined by the method of Rosenthal (1987). The amount of CAT transcription was normalized to β-galactosidase transcription.

### Statistical analysis

Statistical analyses were performed using StatView Statistical Analysis software (SAS Institute, San Francisco, CA, USA). Differences between group mean values were determined by a one-factor analysis of variance (ANOVA), followed by Fisher PSLD post-hoc analysis for pair wise comparison of means.

### RESULTS

### Effect of galangin on the metabolism of [$^3$H]DMBA

MCF-7 cells were incubated with [$^3$H]DMBA in the presence or absence of galangin for 24 h and the catabolism of DMBA was determined by thin-layer chromatography. After 24 h, 65 ± 2.1% (3.1 ± 0.1 nmoles of DMBA per flask per 24 h) of the parent compound had been converted to metabolites in untreated cells. Galangin caused a dose-dependent inhibition of [$^3$H]DMBA catabolism (Figure 1).

The effect of galangin on the formation of adducts between metabolites of DMBA and DNA in MCF-7 cells was examined. In control cultures, exposure to 0.1 μg ml$^{-1}$ [$^3$H]DMBA for 8 h resulted in the formation of 3035 ± 168 fmoles adducts per mg DNA. Exposure of the cells to [$^3$H]DMBA in the presence of galangin inhibited DMBA-DNA adduct formation in a dose-dependent manner (Figure 2).

We measured the growth of MCF-7 cells after 3 days of incubation with increasing concentrations of DMBA in the presence or

 © Cancer Research Campaign 1999

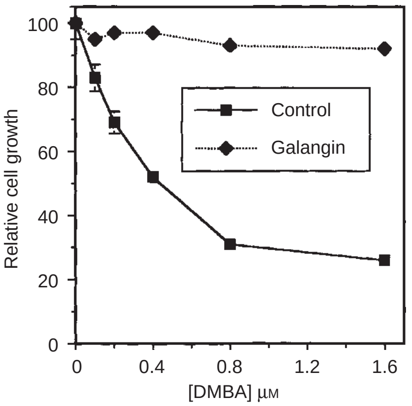

**Figure 3** Effect of galangin on DMBA-induced cytotoxicity. MCF-7 cells were plated out at 25 000 cells per well in 24-well plates. After 24 h, the medium was changed with medium containing the indicated concentrations of DMBA and DMSO (vehicle control) or 5 μM galangin. After 3 days cell growth was measured with sulphorhodamine. Each point represents the mean of 4 determinations ± s.e. Cell growth was significantly different in galangin-treated cultures beginning at 100 nM DMBA ($P < 0.05$)

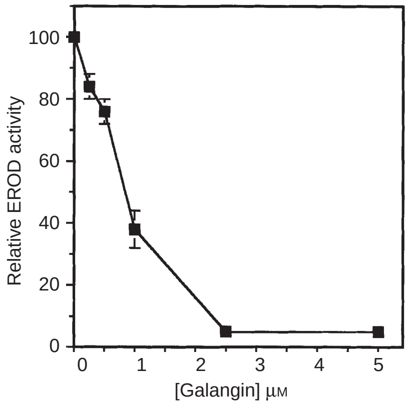

**Figure 4** Effect of galangin on DMBA-induced CYP1A1 activity in intact cells. MCF-7 cells were treated with 1 μM DMBA in the presence of the indicated concentrations of galangin for 24 h. CYP1A1 activity was measured by EROD assay in intact cells as described. EROD activity in control cultures was 4.01 ± 0.12 pmoles per min for each well. Each point represents the mean of 4 determinations ± s.e. EROD activity in all galangin-treated samples was statistically different from controls ($P < 0.05$)

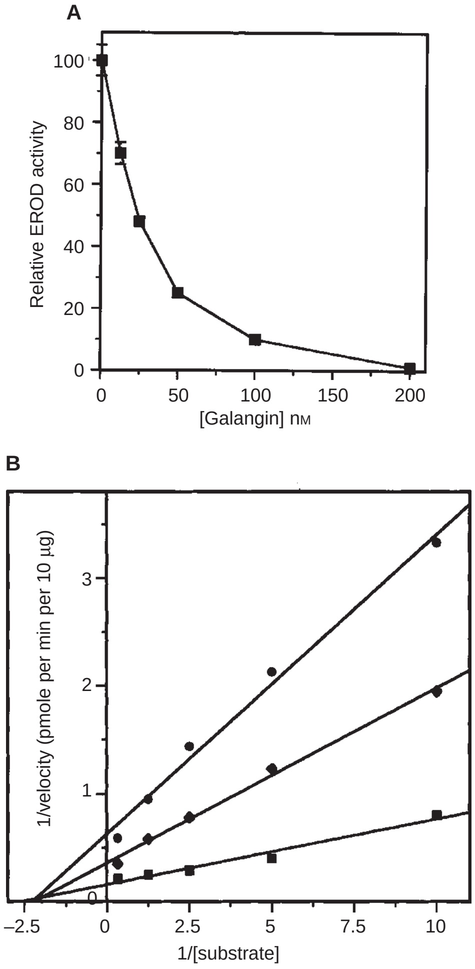

**Figure 5** Effect of galangin on microsomal CYP1A1 activity. Microsomes were isolated from MCF-7 cells treated with 1 μM DMBA. (**A**) The CYP1A1 activity in 10 μg of microsomes was measured by EROD assay in the presence of 400 nM ETRF and the indicated concentrations of galangin. Each point represents the average of 4 determinations ± s.e. EROD activity in controls was 2.33 ± 0.1 pmoles per min per 10 μg. EROD activity in all galangin-treated samples was statistically different from controls ($P < 0.05$). (**B**) EROD activity in 10 μg of microsomes was measured in the presence of 0 (□), 25 (◇), or 50 nM (○) galangin and 100–1600 nM ETRF and a double-reciprocal (Lineweaver-Burk) plot was generated. Each point represents the mean of 4 determinations absence of 5 μM galangin. Galangin completely inhibited DMBA-induced cytotoxicity at the concentrations tested (Figure 3). Galangin at the tested concentration had no effect on cell growth by itself (data not shown).

### Effect of galangin on CYP1A1 activity

We measured the activity of CYP1A1 using the EROD assay, which is specific for the CYP1A1 enzyme family. There was no EROD activity in MCF-7 cells in the absence of DMBA treatment. Treatment of cells with DMBA caused a dose-dependent increase in EROD activity (data not shown). We measured the EROD activity in intact cells which had been exposed to 1 μM DMBA in the presence of galangin. Galangin caused a dose-dependent inhibition of EROD activity with an $IC_{50}$ of less than 1 μM (Figure 4).

The EROD activity in microsomes isolated from cells treated with 1 μM DMBA was potently inhibited by galangin in a dose-dependent manner, with an $IC_{50}$ of approximately 30 nM (Figure 5A). Microsomal EROD activity with or without galangin was also measured in the presence of different substrate concentration and the kinetics of enzyme inhibition by galangin were analysed by double-reciprocal (Lineweaver-Burk) analysis (Figure 5B). The $V_{max}$ of the enzyme shifted from 5.55 pmoles per min per 10 μg microsomes in the absence of galangin to 2.56 or 1.67 pmoles per min per 10 μg in the presence of 25 or 50 nM galangin, while the $K_m$ remained unchanged, indicating a non-competitive type of inhibition.

### Effect of galangin on *CYP1A1* expression

Treatment of MCF-7 cells with 1 nM of the known AhR ligand TCDD for 6 h resulted in a 40-fold increase in CYP1A1 mRNA compared to DMSO control, as determined by semi-quantitative RT-PCR. This increase was inhibited by galangin in a dose-dependent manner (Figure 6A). Treatment of the cells with 250 nM

© Cancer Research Campaign 1999

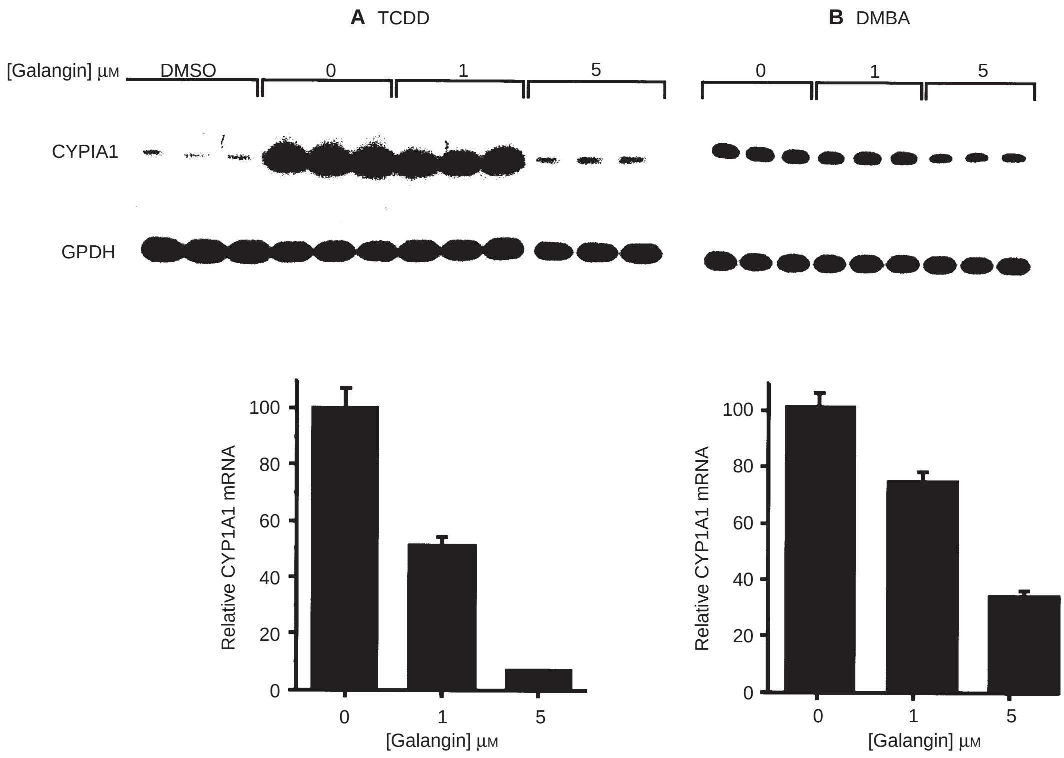

**Figure 6** Effect of galangin on the induction of CYP1A1 mRNA by TCDD or DMBA. MCF-7 cells were treated with 10 nM TCDD or 250 nM DMBA in the presence of the indicated concentrations of galangin for 6 h. Control was treated with DMSO only. RT-PCR was performed, the product was subjected to electrophoresis, and the gel was analysed and quantified with a phosphoimager. For graph, CYP1A1 mRNA was normalized to GPDH mRNA. $n = 3 \pm$ s.e. The level of CYP1A1 mRNA in all galangin-treated samples was statistically different from the TCDD- or DMBA-treated cultures ($P < 0.05$)

DMBA resulted in a tenfold increase in CYP1A1 mRNA, which was also inhibited by galangin (Figure 6B).

The effect of galangin on the transcription of a CAT reporter vector controlled by the *CYP1A1* promoter was determined by transient transfection studies. Treatment of transfected cells for 6 h with 1 nM TCDD caused a sevenfold increase in CAT transcription. This was inhibited in the presence of galangin in a dose-dependent manner (Figure 7). DMBA at 250 nM induced a threefold increase in transcription that was also inhibited by galangin.

Treatment of MCF-7 cells with galangin alone for 24 h resulted in a dose-dependent increase in CYP1A1 mRNA (Figure 8). This increase was blocked by the RNA polymerase inhibitor actinomycin D, indicating that the increase in mRNA was the result of transcriptional activation (data not shown).

### DISCUSSION

Current cancer prevention strategy is based on the growing awareness of the powerful anti-carcinogenic activities of plant-based diets. The central tenet of this strategy is that minor dietary constituents inhibit carcinogenesis through different mechanisms of action. The steps between exposure to a procarcinogen and the transformation of a normal cell to a cancer cell begin with the activation of the procarcinogen by cytochrome $P_{450}$ enzyme(s). This generates metabolites which may be converted to easily excreted forms by Phase II enzymes, but also generates epoxides which are highly electrophilic and carcinogenic, capable of reacting with DNA and causing mutations. One mechanism of action of the so-called ‘blocking’ type of chemopreventive agent, as classified by Wattenburg (1985), is the inhibition of the procarcinogen activation step. In the present study we have investigated the effect of the dietary flavonoid galangin on the carcinogen activation pathway mediated by the AhR. Galangin is of particular interest because its effects on other in vitro mechanisms relevant to chemoprevention has been extensively studied (Critchfield et al, 1994; Eaton et al, 1996; Kao et al, 1998), but there is, to our knowledge, no study which has examined its effect on the AhR and the pathway it regulates.

We assessed the effect of galangin on the activation of DMBA. Galangin inhibited the catabolic breakdown of DMBA (Figure 1). Since the parent compound does not react with DNA, we hypothesized that galangin would decrease DMBA-DNA adduct formation, which proved true (Figure 2). In the tissue culture setting, DMBA inhibits cell growth because of DNA adduct formation. We reasoned that decreased adduct formation would therefore reduce the cytotoxicity of DMBA in vitro. In fact, at the concentration tested, galangin completely abolished the cytotoxic effect of DMBA (Figure 3). Galangin itself was not cytotoxic at the tested concentration. These experiments indicate that galangin inhibits the activation of DMBA to genotoxic metabolites. There is, to our knowledge, no study assessing the physiologically relevant concentrations that galangin may achieve, but other flavonoids have been measured in the plasma and some tissues of humans in the range of concentrations used in this study (Morton et al, 1997; Hollman and Katan, 1998). Moreover, these concentrations correspond to plasma concentrations found in rats fed with a flavonoid-enriched diet (Manach et al, 1995).

© Cancer Research Campaign 1999

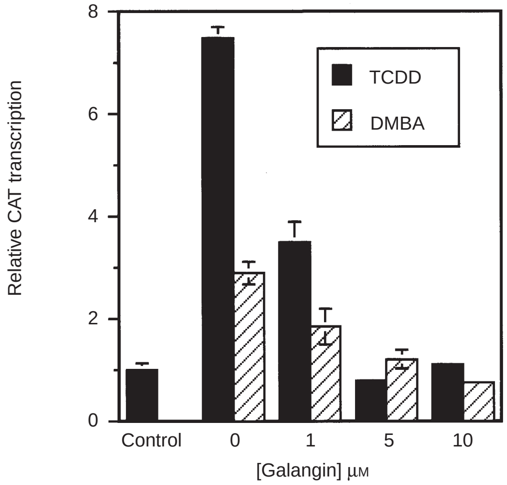

**Figure 7** Effect of galangin on TCDD- or DMBA-induced CAT transcription. MCF-7 cells were transfected with an aryl hydrocarbon-responsive CAT reporter vector containing the full length *CYP1A1* promoter and a β-galactosidase vector as described in Materials and Methods. Cells were treated with 1 nM TCDD or 250 nM DMBA for 6 h in the presence of DMSO (control) or the indicated concentrations of galangin. The amount of CAT transcription was normalized to the amount of β-galactosidase transcription. $n = 4 \pm$ s.e. The amount of CAT transcription in galangin-treated cultures was statistically different from TCDD- or DMBA-treated cultures ($P < 0.05$)

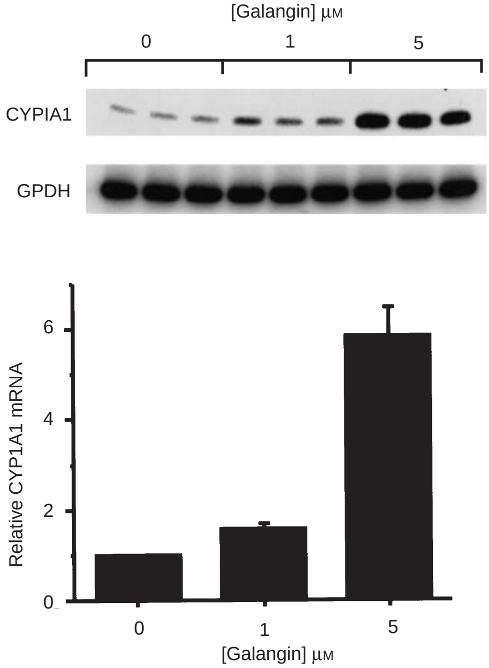

**Figure 8** Effect of galangin on CYP1A1 mRNA. MCF-7 cells were treated with the indicated concentrations of galangin for 24 h. For graph, CYP1A1 mRNA was normalized to GPDH mRNA ± s.e. The amount of CYP1A1 mRNA was statistically significant from controls in cultures treated with 5 μM, but not 1 μM, galangin

We next examined the mechanism(s) of galangin's action. The activity of CYP1A1 was measured by EROD assay, a specific indicator of the CYP1A1 bioactivation capabilities. Under conditions of AhR activation, the major DMBA activating isozyme in MCF-7 cells is CYP1A1 (Christou et al, 1994). These cells also express CYP1B1, but it is unclear whether this isozyme can metabolize DMBA (Shimada et al, 1997), or whether CYP1B1 possess significant EROD activity (Dohr et al, 1995; Shimada et al, 1997). Co-treatment of the cells with DMBA and increasing concentrations of galangin resulted in a dose-dependent inhibition of DMBA-induced EROD activity in intact cells (Figure 4). We could not determine, from this experiment, whether galangin's effect was as a result of inhibition at the enzyme level or through a disruption of the signal transduction pathway leading to the transcriptional activation of the *CYP1A1* gene. We therefore performed EROD assays on microsomes isolated from DMBA-treated cells. Galangin proved to be a potent inhibitor of microsomal EROD activity (Figure 5A). Previous studies have shown that the related flavonol quercetin inhibits EROD activity in rat hepatic microsomes in a competitive fashion (Sousa and Marletta, 1985), a result we confirmed in MCF-7 microsomes (data not shown). Analysis of the kinetics of inhibition by galangin, however, revealed that galangin inhibits EROD activity in a non-competitive manner (Figure 5B). Thus, the interaction of galangin with CYP1A1 is different than the structurally similar quercetin.

DMBA causes the transcriptional activation of the *CYP1A1* gene through the AhR. Although the results of the microsomal EROD assays indicate that direct enzyme inhibition by galangin can account for the decrease in DMBA-induced EROD activity in intact cells, we also tested the hypothesis that galangin may disrupt the transciptional activation of *CYP1A1*. When MCF-7 cells were treated with 250 nM DMBA for 6 h, there was an approximately tenfold increase in CYP1A1 mRNA compared to DMSO control. Co-treatment with galangin caused a dose-dependent inhibition of the DMBA-induced increase in CYP1A1 mRNA (Figure 6B). Galangin also inhibited the increase in CYP1A1 mRNA by the prototypical AhR ligand TCDD (Figure 6A). Galangin also inhibited the DMBA- or TCDD-induced transcription of CAT in a reporter vector controlled by the *CYP1A1* promoter (Figure 7). We also observed that treatment of the cells with galangin alone for a longer period of time (24 h) could induce a mild increase (compared to DMBA or TCDD) in CYP1A1 mRNA (Figure 8). This increase was blocked by the RNA polymerase inhibitor actinomycin D, which prevents de novo RNA synthesis resulting from transcriptional activation (data not shown). These results are consistent with the hypothesis that galangin is a weak ligand of the AhR and is able to compete for the AhR with traditional activators of the receptor, resulting overall in a decrease in CYP1A1 mRNA caused by other, more potent AhR ligands. The induction of CYP1A1 mRNA and the inhibition of CYP1A1 EROD activity by galangin may indicate that it is a natural substrate of this metabolic pathway in MCF-7 cells, but this was not examined in the present study.

Known ligands of the AhR are, for the most part, man-made chemicals; natural ligands have been postulated but, with the exception of indolo[2,3]-carbazole (Jellinck et al, 1993), remain unidentified. We have hypothesized elsewhere (Ciolino et al, 1998) that dietary polyphenolic compounds are natural ligands of the AhR and galangin appears to be such a compound. These experiments demonstrate that galangin inhibits carcinogen activation in MCF-7 cells at two levels: through direct inhibition of CYP1A1 enzyme activity and by inhibiting the increase in *CYP1A1* transcription caused by AhR ligands. There is no study, to our knowledge, on the chemopreventive effect of galangin in animal models of carcinogenesis. Based on our data, galangin may be a promising candidate for in vivo chemoprevention studies.

© Cancer Research Campaign 1999 

### ACKNOWLEDGEMENTS

The authors wish to thank Dr Thomas Barlow and Dr Anthony Dipple for their help in measuring DMBA metabolism, and Dr Robert Clarke for his critical reading of this manuscript.

### REFERENCES

Bradford M (1976) A rapid and sensitive method for the quantitation of microgram quantities of protein utilizing the principle of protein-dye binding. *Anal Biochem* **72**: 248–254

Buening M, Chan R, Huang M, Fortner J, Wood A and Conney A (1981) Activation and inhibition of benzo(*a*)pyrene and aflatoxin B1 metabolism in human liver microsomes by naturally occurring flavonoids. *Cancer Res* **41**: 67–72

Canivenc-Lavier M, Vernevaut M, Totis M, Siess M, Magdalou J and Suschetet M (1996) Comparative effects of flavonoids and model inducers on drug metabolizing enzymes in rat liver. *Toxicology* **114**: 19–27

Christou M, Savas U, Spink D, Gierthy J and Jefcoate C (1994) Co-expression of human CYP1A1 and a human analog of cytochrome $P_{450}$-EF in response to 2,3,7,8-cells. tetrachlorodibenzo-*p*-dioxin in the human mammary carcinoma-derived MCF-7 cell. *Carcinogenesis* **15**: 725–732

Ciolino H, Daschner P, Wang T and Yeh G (1998) The effect of curcumin on the aryl hydrocarbon receptor and cytochrome $P_{450}$ 1A1 in MCF-7 human breast carcinoma cells. *Biochem Pharmacol* **56**: 197–206

Critchfield J, Welsh C, Phang J and Yeh G (1994) Modulation of adriamycin accumulation and efflux by flavonoids in HCT-15 colon cells. Activation of P-glycoprotein as a putative mechanism. *Biochem Pharmacol* **48**: 1437–1445

Csokay B, Prajda N, Weber G and Olah E (1997) Molecular mechanisms in the antiproliferative action of quercetin. *Life Sci* **60**: 2157–2163

Dipple A (1995) DNA adducts of chemical carcinogens. *Carcinogenesis* **16**: 437–441

Dohr O, Vogel C and Abel J (1995) Different response of 2,3,7,8-tetrachlorodibenzo-*p*-dioxin (TCDD)-sensitive genes in human breast cancer MCF-7 and MDA-MB 231 cells. *Arch Biochem Biophys* **321**: 405–412

Eaton E, Walle U, Lewis A, Hudson T, Wilson A and Walle T (1996) Flavonoids, potent inhibitors of the human P-form phenolsulfotransferase. Potential role in drug metabolism and chemoprevention. *Drug Metab Dispo* **24**: 232–237

Formica J and Regelson W (1995) Review of the biology of quercetin and related bioflavonoids. *Food Chem Toxicol* **33**: 1061–1080

Hollman P and Katan M (1997) Absorption, metabolism and health effects of dietary flavonoids in man. *Biomed Pharmacother* **51**: 305–310

Hollman P and Katan M (1998) Bioavailability and health effects of dietary flavonols in man. *Arch Toxicol Suppl* **20**: 237–248

Hollman P, van Trijp J, Mengelers M, de Vries J and Katan M (1997) Bioavailability of the dietary antioxidant flavonol quercetin in man. *Cancer Lett* **114**: 139–140

Huggins C, Grand L and Brillantes F (1961) Mammary cancer induced by a single feeding of polynuclear hydrocarbons, and its suppression. *Nature* **189**: 204–207

Jellinck P, Forkert P, Riddick D, Okey A, Michnovicz J and Bradlow H (1993) Ah receptor binding properties of indole carbinols and induction of hepatic estradiol hydroxylation. *Biochem Pharmacol* **45**: 1129–1136

Kao Y, Zhou C, Sherman M, Laughton C and Chen S (1998) Molecular basis of the inhibition of human aromatase (estrogen synthetase) by flavone and isoflavone phytoestrogens: a site-directed mutagenesis study. *Environ Health Perspect* **106**: 85–92

Kennedy S and Jones S (1994) Simultaneous measurement of cytochrome $P_{450}$ 1A catalytic activity and total protein concentration with a fluorescence plate reader. *Anal Biochem* **222**: 217–223

Kono Y, Kobayashi K, Tagawa S, Adachi K, Ueda A, Sawa Y and Shibata H (1997) Antioxidant activity of polyphenolics in diets. Rate constants of reactions of chlorogenic acid and caffeic acid with reactive species of oxygen and nitrogen. *Biochim Biophys Acta* **1335**: 335–342

Kuhnau J (1976) The flavonoids. A class of semi-essential food components: their role in human nutrition. *World Rev Nutr Diet* **24**: 117–191

Lamartiniere C, Moore J, Brown N, Thompson R, Hardin M and Barnes S (1995) Genistein suppresses mammary cancer in rats. *Carcinogenesis* **16**: 2833–2840

Larsen MC, Angus WG, Brake PB, Eltom SE, Sukow KA and Jefcoate CR (1998) Characterization of CYP1B1 and CYP1A1 expression in human mammary epithelial cells: role of the aryl hydrocarbon receptor in polycyclic aromatic hydrocarbon metabolism. *Cancer Res* **58**: 2366–2374

Lubinski J, Flint O and Durham S (1994) In vivo and in vitro studies of rat liver cytochrome $P_{450}$ induction: II. In vitro induction by phenobarbitol and 3-methylcholanthrene measured in an automated 24-well plate assay for cytochrome $P_{450}$-dependent activity (pentoxyresorufin-*O*-deethylase and ethoxyresorufin-*O*-deethylase). *In Vitro Toxicol* **7**: 13–23

Manach C, Morand C, Texier O, Favier M, Agullo G, Demigne C, Regerat F and Remsy C (1995) Quercetin metabolites in plasma of rats fed diets containing rutin or quercetin. *J Nutr* **125**: 1911–1922

Miller S, Dykes D and Polesky H (1988) A simple salting out procedure for extracting DNA from human nucleated cells. *Nucleic Acids Res* **16**: 1215

Mirzoeva O and Calder P (1996) The effect of propolis and its components on eicosanoid production during the inflammatory response. *Prostaglandins Leukot Essent Fatty Acids* **55**: 441–449

Moon JY, Lee DW and Park KH (1998) Inhibition of 7-ethoxycoumarin *O*-deethylase activity in rat liver microsomes by naturally occurring flavonoids: structure-activity relationships. *Xenobiotica* **28**: 117–126

Moore M, Wang X, Lu Y, Wormke M, Craig A, Gerlach J, Burghardt R, Barhoumi R and Safe S (1994) Benzo[*a*]pyrene-resistant MCF-7 human breast cancer cells. A unique aryl hydrocarbon-nonresponsive clone. *J Biol Chem* **269**: 11751–11759

Morton M, Chan P, Cheng C, Blacklock N, Matos-Ferreira A, Abranches-Monteiro L, Correia R, Lloyd S and Griffiths K (1997) Lignans and isoflavonoids in plasma and prostatic fluid in men: samples from Portugal, Hong Kong, and the United Kingdom. *Prostate* **32**: 122–128

Noda Y, Anzai K, Mori A, Kohno M, Shinmei M and Packer L (1997) Hydroxyl and superoxide anion radical scavenging activities of natural source antioxidants using the computerized JES-FR30 ESR spectrometer system. *Biochem Mol Biol Int* **42**: 35–44

Rosenthal N (1987) Identification of regulatory elements of cloned genes with functional assays. *Methods Enzymol* **152**: 704

Rowlands J and Gustafsson J (1997) Aryl hydrocarbon receptor-mediated signal transduction. *Crit Rev Toxicol* **27**: 109–134

Rubinstein L, Shoemaker R, Paull K, Simon R, Tosin S, Skehan P, Scudiero D, Monks A and Boyd M (1990) Comparison of in vitro anticancer-drug-screening data generated with a tetrazolium assay versus a protein assay against a diverse panel of human tumor cell lines. *J Natl Cancer Inst* **82**: 1113–1118

Shimada T, Gillam EM, Sutter TR, Strickland PT, Guengerich FP and Yamazaki H (1997) Oxidation of xenobiotics by recombinant human cytochrome P450 1B1 *Drug Metab Dispos* **25**: 617–622

Siess M, Leclerc J, Canivenc-Lavier M, Rat P and Suschetet M (1995) Heterogenous effects of natural flavonoids on monooxygenase activities in human and rat liver microsomes. *Toxicol Appl Pharmacol* **130**: 73–78

So F, Guthrie N, Chambers A, Moussa M and Carroll K (1996) Inhibition of human breast cancer cell proliferation and delay of mammary tumorigenesis by flavonoids and citrus juices. *Nutr Cancer* **26**: 167–181

So F, Guthrie N, Chambers A and Carroll K (1997) Inhibition of proliferation of estrogen receptor-positive MCF-7 human breast cancer cells by flavonoids in the presence and absence of excess estrogen. *Cancer Lett* **112**: 127–133

Sogawa K, Fujisawa-Sehara A, Yamane M and Fujii-Kuriyama Y (1986) Location of regulatory elements responsible for drug induction in the rat cytochrome $P_{450c}$ gene. *Proc Natl Acad Sci USA* **83**: 8044–8048

Sousa R and Marletta M (1985) Inhibition of cytochrome $P_{450}$ activity in rat liver microsomes by the naturally occurring flavonoid, quercetin. *Arch Biochem Biophys* **240**: 345–357

Steinmetz K and Potter J (1996) Vegetables, fruit, and cancer prevention: a review. *J Am Diet Assoc* **96**: 1027–1039

Tsyrlov IB, Mikhailenko VM and Gelboin HV (1994) Isozyme- and species-specific susceptibility of cDNA-expressed CYP1A $P_{450}$s to different flavonoids. *Biochim Biophys Acta* **1205**: 325–335

Verma A, Johnson J, Gould M and Tanner M (1988) Inhibition of 7,12-dimethylbenz-[*a*]-anthracene- and *N*-nitrosomethylurea-induced rat mammary cancer by dietary flavonol quercetin. *Cancer Res* **48**: 5754–5758

Wang X, Thomsen J, Santostefano M, Rosengren R, Safe S and Perdew G (1995) Comparative properties of the nuclear aryl hydrocarbon (Ah) receptor complex from several human cell lines. *Eur J Pharmacol* **293**: 191–205

Wattenberg L (1985) Chemoprevention of cancer. *Cancer Res* **45**: 1–8

*© Cancer Research Campaign 1999*

## References

[PDF_00603] Bradford M. M. (1976). A rapid and sensitive method for the quantitation of microgram quantities of protein utilizing the principle of protein-dye binding.. Anal Biochem.

[PDF_00606] Buening M. K., Chang R. L., Huang M. T., Fortner J. G., Wood A. W., Conney A. H. (1981). Activation and inhibition of benzo(a)pyrene and aflatoxin B1 metabolism in human liver microsomes by naturally occurring flavonoids.. Cancer Res.

[PDF_00609] Canivenc-Lavier M. C., Vernevaut M. F., Totis M., Siess M. H., Magdalou J., Suschetet M. (1996). Comparative effects of flavonoids and model inducers on drug-metabolizing enzymes in rat liver.. Toxicology.

[PDF_00612] Christou M., Savas U., Spink D. C., Gierthy J. F., Jefcoate C. R. (1994). Co-expression of human CYP1A1 and a human analog of cytochrome P450-EF in response to 2,3,7,8-tetrachloro-dibenzo-p-dioxin in the human mammary carcinoma-derived MCF-7 cells.. Carcinogenesis.

[PDF_00617] Ciolino H. P., Daschner P. J., Wang T. T., Yeh G. C. (1998). Effect of curcumin on the aryl hydrocarbon receptor and cytochrome P450 1A1 in MCF-7 human breast carcinoma cells.. Biochem Pharmacol.

[PDF_00621] Critchfield J. W., Welsh C. J., Phang J. M., Yeh G. C. (1994). Modulation of adriamycin accumulation and efflux by flavonoids in HCT-15 colon cells. Activation of P-glycoprotein as a putative mechanism.. Biochem Pharmacol.

[PDF_00624] Csokay B., Prajda N., Weber G., Olah E. (1997). Molecular mechanisms in the antiproliferative action of quercetin.. Life Sci.

[PDF_00626] Dipple A. (1995). DNA adducts of chemical carcinogens.. Carcinogenesis.

[PDF_00628] Döhr O., Vogel C., Abel J. (1995). Different response of 2,3,7,8-tetrachlorodibenzo-p-dioxin (TCDD)-sensitive genes in human breast cancer MCF-7 and MDA-MB 231 cells.. Arch Biochem Biophys.

[PDF_00631] Eaton E. A., Walle U. K., Lewis A. J., Hudson T., Wilson A. A., Walle T. (1996). Flavonoids, potent inhibitors of the human P-form phenolsulfotransferase. Potential role in drug metabolism and chemoprevention.. Drug Metab Dispos.

[PDF_00634] Formica J. V., Regelson W. (1995). Review of the biology of Quercetin and related bioflavonoids.. Food Chem Toxicol.

[PDF_00642] HUGGINS C., GRAND L. C., BRILLANTES F. P. (1961). Mammary cancer induced by a single feeding of polymucular hydrocarbons, and its suppression.. Nature.

[PDF_00636] Hollman P. C., Katan M. B. (1997). Absorption, metabolism and health effects of dietary flavonoids in man.. Biomed Pharmacother.

[PDF_00638] Hollman P. C., Katan M. B. (1998). Bioavailability and health effects of dietary flavonols in man.. Arch Toxicol Suppl.

[PDF_00640] Hollman P. C., van Trijp J. M., Mengelers M. J., de Vries J. H., Katan M. B. (1997). Bioavailability of the dietary antioxidant flavonol quercetin in man.. Cancer Lett.

[PDF_00644] Jellinck P. H., Forkert P. G., Riddick D. S., Okey A. B., Michnovicz J. J., Bradlow H. L. (1993). Ah receptor binding properties of indole carbinols and induction of hepatic estradiol hydroxylation.. Biochem Pharmacol.

[PDF_00647] Kao Y. C., Zhou C., Sherman M., Laughton C. A., Chen S. (1998). Molecular basis of the inhibition of human aromatase (estrogen synthetase) by flavone and isoflavone phytoestrogens: A site-directed mutagenesis study.. Environ Health Perspect.

[PDF_00651] Kennedy S. W., Jones S. P. (1994). Simultaneous measurement of cytochrome P4501A catalytic activity and total protein concentration with a fluorescence plate reader.. Anal Biochem.

[PDF_00655] Kono Y., Kobayashi K., Tagawa S., Adachi K., Ueda A., Sawa Y., Shibata H. (1997). Antioxidant activity of polyphenolics in diets. Rate constants of reactions of chlorogenic acid and caffeic acid with reactive species of oxygen and nitrogen.. Biochim Biophys Acta.

[PDF_00659] Kühnau J. (1976). The flavonoids. A class of semi-essential food components: their role in human nutrition.. World Rev Nutr Diet.

[PDF_00661] Lamartiniere C. A., Moore J. B., Brown N. M., Thompson R., Hardin M. J., Barnes S. (1995). Genistein suppresses mammary cancer in rats.. Carcinogenesis.

[PDF_00663] Larsen M. C., Angus W. G., Brake P. B., Eltom S. E., Sukow K. A., Jefcoate C. R. (1998). Characterization of CYP1B1 and CYP1A1 expression in human mammary epithelial cells: role of the aryl hydrocarbon receptor in polycyclic aromatic hydrocarbon metabolism.. Cancer Res.

[PDF_00674] Manach C., Morand C., Texier O., Favier M. L., Agullo G., Demigné C., Régérat F., Rémésy C. (1995). Quercetin metabolites in plasma of rats fed diets containing rutin or quercetin.. J Nutr.

[PDF_00677] Miller S. A., Dykes D. D., Polesky H. F. (1988). A simple salting out procedure for extracting DNA from human nucleated cells.. Nucleic Acids Res.

[PDF_00679] Mirzoeva O. K., Calder P. C. (1996). The effect of propolis and its components on eicosanoid production during the inflammatory response.. Prostaglandins Leukot Essent Fatty Acids.

[PDF_00682] Moon J. Y., Lee D. W., Park K. H. (1998). Inhibition of 7-ethoxycoumarin O-deethylase activity in rat liver microsomes by naturally occurring flavonoids: structure-activity relationships.. Xenobiotica.

[PDF_00685] Moore M., Wang X., Lu Y. F., Wormke M., Craig A., Gerlach J. H., Burghardt R., Barhoumi R., Safe S. (1994). Benzo[a]pyrene-resistant MCF-7 human breast cancer cells. A unique aryl hydrocarbon-nonresponsive clone.. J Biol Chem.

[PDF_00689] Morton M. S., Chan P. S., Cheng C., Blacklock N., Matos-Ferreira A., Abranches-Monteiro L., Correia R., Lloyd S., Griffiths K. (1997). Lignans and isoflavonoids in plasma and prostatic fluid in men: samples from Portugal, Hong Kong, and the United Kingdom.. Prostate.

[PDF_00693] Noda Y., Anzai K., Mori A., Kohno M., Shinmei M., Packer L. (1997). Hydroxyl and superoxide anion radical scavenging activities of natural source antioxidants using the computerized JES-FR30 ESR spectrometer system.. Biochem Mol Biol Int.

[PDF_00697] Rosenthal N. (1987). Identification of regulatory elements of cloned genes with functional assays.. Methods Enzymol.

[PDF_00699] Rowlands J. C., Gustafsson J. A. (1997). Aryl hydrocarbon receptor-mediated signal transduction.. Crit Rev Toxicol.

[PDF_00701] Rubinstein L. V., Shoemaker R. H., Paull K. D., Simon R. M., Tosini S., Skehan P., Scudiero D. A., Monks A., Boyd M. R. (1990). Comparison of in vitro anticancer-drug-screening data generated with a tetrazolium assay versus a protein assay against a diverse panel of human tumor cell lines.. J Natl Cancer Inst.

[PDF_00705] Shimada T., Gillam E. M., Sutter T. R., Strickland P. T., Guengerich F. P., Yamazaki H. (1997). Oxidation of xenobiotics by recombinant human cytochrome P450 1B1.. Drug Metab Dispos.

[PDF_00708] Siess M. H., Leclerc J., Canivenc-Lavier M. C., Rat P., Suschetet M. (1995). Heterogenous effects of natural flavonoids on monooxygenase activities in human and rat liver microsomes.. Toxicol Appl Pharmacol.

[PDF_00714] So F. V., Guthrie N., Chambers A. F., Carroll K. K. (1997). Inhibition of proliferation of estrogen receptor-positive MCF-7 human breast cancer cells by flavonoids in the presence and absence of excess estrogen.. Cancer Lett.

[PDF_00711] So F. V., Guthrie N., Chambers A. F., Moussa M., Carroll K. K. (1996). Inhibition of human breast cancer cell proliferation and delay of mammary tumorigenesis by flavonoids and citrus juices.. Nutr Cancer.

[PDF_00717] Sogawa K., Fujisawa-Sehara A., Yamane M., Fujii-Kuriyama Y. (1986). Location of regulatory elements responsible for drug induction in the rat cytochrome P-450c gene.. Proc Natl Acad Sci U S A.

[PDF_00720] Sousa R. L., Marletta M. A. (1985). Inhibition of cytochrome P-450 activity in rat liver microsomes by the naturally occurring flavonoid, quercetin.. Arch Biochem Biophys.

[PDF_00724] Steinmetz K. A., Potter J. D. (1996). Vegetables, fruit, and cancer prevention: a review.. J Am Diet Assoc.

[PDF_00726] Tsyrlov I. B., Mikhailenko V. M., Gelboin H. V. (1994). Isozyme- and species-specific susceptibility of cDNA-expressed CYP1A P-450s to different flavonoids.. Biochim Biophys Acta.

[PDF_00730] Verma A. K., Johnson J. A., Gould M. N., Tanner M. A. (1988). Inhibition of 7,12-dimethylbenz(a)anthracene- and N-nitrosomethylurea-induced rat mammary cancer by dietary flavonol quercetin.. Cancer Res.

[PDF_00733] Wang X., Thomsen J. S., Santostefano M., Rosengren R., Safe S., Perdew G. H. (1995). Comparative properties of the nuclear aryl hydrocarbon (Ah) receptor complex from several human cell lines.. Eur J Pharmacol.

[PDF_00736] Wattenberg L. W. (1985). Chemoprevention of cancer.. Cancer Res.

